# Relationship Between Echo-Intensity Bands of the Vastus Lateralis and Rectus Femoris Muscles and Torque Parameters of Knee Extensors in Soccer Players

**DOI:** 10.3390/jfmk11030261

**Published:** 2026-06-30

**Authors:** Maria Rita dos Santos Lara, Silas Nery de Oliveira, Luiz Henrique Rufino Batista, Silvio Assis de Oliveira-Junior, Eduardo Feijó da Rocha, Rodolfo André Dellagrana, Mateus Rossato

**Affiliations:** 1 Human Laboratory Performance, Faculty of Physical Education and Physiotherapy, Federal University of Amazonas, Manaus 69080-900, AM, Brazil; mariaritalara2402@gmail.com (M.R.d.S.L.); nerysilas@gmail.com (S.N.d.O.); henrique.batista@ufam.edu.br (L.H.R.B.); mateusrossato@ufam.edu.br (M.R.); 2 Graduate Program in Movement Sciences, Federal University of Mato Grosso do Sul (UFMS), Campo Grande 79070-900, MS, Brazil; silvio.oliveira-jr@ufms.br (S.A.d.O.-J.); eduardofdr@gmail.com (E.F.d.R.); 3 Department of Physical Education, State University of Ponta Grossa, Ponta Grossa 84030-900, PR, Brazil

**Keywords:** ultrasonography, muscle strength, athletic performance, isokinetic dynamometry, subcutaneous fat

## Abstract

**Background**: Echo intensity (EI) derived from ultrasound imaging is widely used to assess muscle quality and has been proposed as a potential indicator of neuromuscular performance. Recently, EI band analysis has been suggested as an alternative approach to provide additional information beyond mean EI. However, evidence linking EI bands to functional outcomes remains limited. **Methods**: Forty-eight male soccer players (26.2 ± 3.6 years) underwent ultrasound assessment of the vastus lateralis (VL) and rectus femoris (RF) muscles. EI was analyzed as mean values and as pixel distribution across five bands (0–50, 51–100, 101–150, 151–200, and 201–255 A.U.), with correction for subcutaneous adipose thickness. Knee extensor peak concentric torque and total work were assessed using isokinetic dynamometry at 60°/s. Generalized linear regression models were used to examine associations between EI variables and mechanical outcomes. **Results**: No significant differences were observed between limbs for EI mean or EI band distribution in either muscle. The 0–50 A.U. band exhibited the highest pixel percentage for both VL and RF. Mean EI of the RF was negatively associated with peak torque (β = −4.10; 95% CI: −7.35 to −0.86) and total work (β = −3.89; 95% CI: −6.34 to −1.45) of the right knee extensors. No significant associations were found for EI bands or for any EI variables of the VL muscle. In male soccer players, mean EI of the rectus femoris, but not EI band distribution, is associated with knee extensor torque and work output. **Conclusions**: These findings suggest that mean EI remains a more informative indicator of muscle quality related to strength performance than band-based EI analysis in this athletic population.

## 1. Introduction

In the context of physical exercise, echo intensity (EI) obtained from ultrasound images has been used to assess muscle damage, cellular swelling, glycogen content, and muscle quality [1]. EI is related to the pixel density present in each area [2] and can vary from 0 (black) to 255 A.U. (white), with its results traditionally expressed as average values (average EI). Lighter images of muscle tissue are associated with a greater amount of fibrous material and infiltrated adipose tissue [3], while a reduction in EI (darker images) is associated with increased strength [4] and athletic performance [5], suggesting the presence of greater contractile content.

Recently, some authors have proposed a new interpretation of EI that involves grouping pixel concentrations by band [6,7,8]. According to these authors, band separation could provide additional information on muscle composition assessed by ultrasound. However, a larger body of evidence is needed to confirm or refute these findings. Pinto and Pinto [7] compared bands every 50 pixels of the rectus femoris (RF) in young adults and older adults. The authors observed that EI 0–50 A.U. decreased significantly, while the others increased with age. The authors concluded that analyzing EI by bands may be a promising approach to understanding specific muscle tissue adaptations to physical training and neuromuscular diseases.

Logeson et al. [6] evaluated the vastus lateralis (VL) of 13 women who maintained knee immobilization for two weeks and ambulated with crutches. The authors separated it into 50-pixel bands and observed that immobilization decreased the percentage of pixels in the 0–50 A.U. band but increased the percentages in the 101–150 A.U. and 151–200 A.U. ranges, indicating a deterioration in muscle quality. Finally, Southall et al. [8] compared the relationships between the mean EI and the single bands of the VL ultrasound signal with fatigue metrics obtained during the performance of 100 isokinetic contractions (120°/s). The authors did not report a significant correlation between mean EI and the fatigue index, or between any of the investigated bands and the fatigue index. The authors concluded that the EI band analysis does not offer different relationships compared to average EI when assessing knee extensor fatigue.

Despite this initial effort, it is worth noting that these studies focused on understanding changes in bands across different age groups [7], rehabilitation [6], and activities involving fatigue [8]. Furthermore, these studies did not account for the thickness of subcutaneous adipose tissue (SAT) in their analyses. Muller and Ye [9] reported that an increase in SAT could underestimate IE values. According to the authors, this occurs because the SAT promotes attenuation of sound-wave propagation. Therefore, the authors proposed an equation to correct IE using the SAT layer. Thus, additional studies are needed to better understand the relationship between the different EI bands and functional capacity, especially in the knee extensor muscles of soccer players, since these muscles are directly involved with actions such as kicking and sprints.

Our initial hypothesis is that participants with a higher pixel density in the darker bands would also exhibit higher torque values and concentric work in the knee extensors. Therefore, the objective of the study was to evaluate the relationship between the IE bands of the VL and RF muscles and the torque and work output between the knee extensors.

## 2. Materials and Methods

### 2.1. Participants

**Figure 1:**
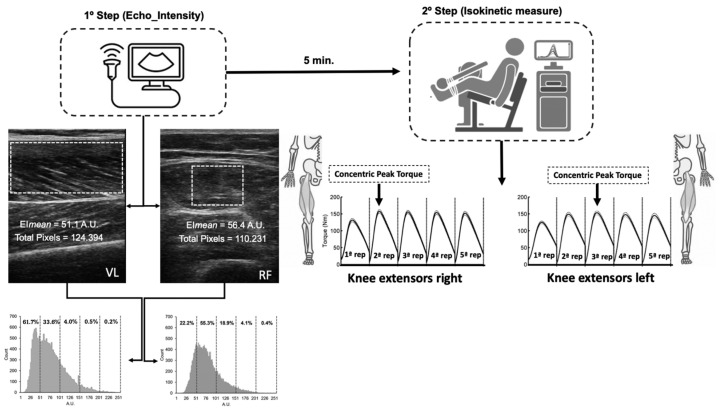
Flowchart of study.

Twenty-two (26.2 ± 3.6 years, 181 ± 6 cm and 77.3 ± 7.9 kg) male soccer players participated in this study, and 19 athletes reported having no right limb as dominant for the kick. The athletes engaged in regular technical, tactical, and physical training and competed at regional and national levels. They reported no history of injury in the preceding six months. The evaluation protocol encompassed: (a) ultrasonography for quadriceps morphology assessment (Left and Right) and (b) isokinetic dynamometry for torque parameter evaluation (Figure 1). Before assessments, an interview was conducted, followed by obtaining informed consent from all participants. This study was approved by the local University Ethics Committee and conducted in accordance with the Declaration of Helsinki. Data collection took place between January and February 2025.

### 2.2. Assessment of Muscle Quality

To assess muscle quality, we used a B-mode ultrasound system (DP-30 Mindray, Shenzhen Mindray Bio-Medical Electronics Co., Ltd., Shenzhen, China) with a linear probe operating at 32 Hz (60 mm, 7.5 MHz, 4.0 cm depth, without a filter). The participant remained in the supine position for 10 min to stabilize body fluids. Muscle quality assessed by echo intensity was determined for the VL and RF muscles. Three images were obtained for each muscle with the subject at rest. The US probe was covered with water-soluble transmission gel and positioned longitudinally to the muscle fibers and perpendicularly to the skin at 50% (RF, VL) of thigh length [7]. The US probe was placed on the skin surface, parallel to the superficial and deep aponeuroses, and aligned with the hyperechoic intramuscular connective tissue of the perimysium. Probe alignment was considered adequate when the superficial and deep aponeuroses were parallel and multiple fascicles could be easily delineated without interruption of the image [10]. All US images were analyzed using ImageJ software (straight line, line color: yellow, version 1.48 v, National Institutes of Health, Bethesda, MD, USA). The histogram produced in ImageJ was exported to a Microsoft Excel spreadsheet to present EI bands in 50 A.U. intervals (0–50, 51–100, 101–150, 150–200, and 201–255 A.U.). The quantity of pixels in each band was then expressed as a percentage of the total pixel content in the EI histogram. Expressing the number of EI pixels in each band as a percentage of the total number allows for normalization of images possessing varying numbers of pixels. This procedure was adapted from Logeson et al. [6]. Subcutaneous adipose thickness (SAT) was quantified using ImageJ^®^, where the mean of eleven measurements captured with the straight-line function was used for the correction of the EI. The distance between the skin and the superficial aponeurosis of the VL was used. The EI values were then corrected for SAT using the following equation created by Muller and Ye [9].

EIcorrected = EImeasured − 5.0054 × SAT [cm] + (38.30836 × SAT [cm])

where EI = echo intensity, and SAT = subcutaneous adipose thickness.

### 2.3. Evaluation of Concentric Torque Parameters

Before the assessments, participants were positioned on an isokinetic dynamometer (Biodex System 4 Pro; Biodex Medical Systems, Shirley, NY, USA) according to the manufacturer’s recommendations for assessing knee extensor and flexor muscles. A familiarization protocol and warm-up were performed (20 submaximal concentric knee extension-flexion repetitions). The isokinetic evaluation protocol was adapted from Rossato et al. [11]. Following the warm-up, participants performed two sets of five concentric knee extension repetitions at 60°/s with a 70° range of motion (30–100°, with 0° = full knee extension). A two-minute interval was given between sets. The torque signal was sampled at 100 Hz and smoothed using a fifth-order, zero-phase, low-pass Butterworth recursive filter with a cut-off frequency of 10 Hz. For analysis, the best concentric peak torque (PTCon) and the total work (Wtotal) of the best set were considered, although both sides were evaluated.

### 2.4. Statistical Analysis

Descriptive statistics are presented as means and standard deviations. Data normality and homogeneity of variance were verified using the Shapiro–Wilk and Levene’s tests, respectively. Inter-limb comparisons (PTCon, Total Work, and EI_normalized_) were performed using paired *t*-tests. To evaluate and compare the percentage of pixels across the echo intensity spectrum, a mixed-design ANOVA was conducted for both the vastus lateralis (VL) and rectus femoris (RF) muscles, using echo intensity bands (0–50, 51–100, 101–150, 151–200, and 201–255 A.U.) as the within-subject factor and limb side (right vs. left) as the between-subjects factor. In the event of significant main effects or interactions, post hoc comparisons with Bonferroni adjustments were applied. Additionally, a generalized linear regression analysis was used to assess the association between the 5 bands and both torque and power output. Effect sizes were calculated using Cohen’s d for paired comparisons and partial eta-squared (ηp^2^) for the ANOVA. Cohen’s d values were interpreted as small (0.20), medium (0.50), or large (≥0.80), whereas ηp^2^ values were classified as small (0.01), medium (0.06), or large (≥0.14). Statistical analyses were performed using IBM SPSS Statistics (Version 27.0; IBM Corp., Armonk, NY, USA).

## 3. Results

**Figure 2:**
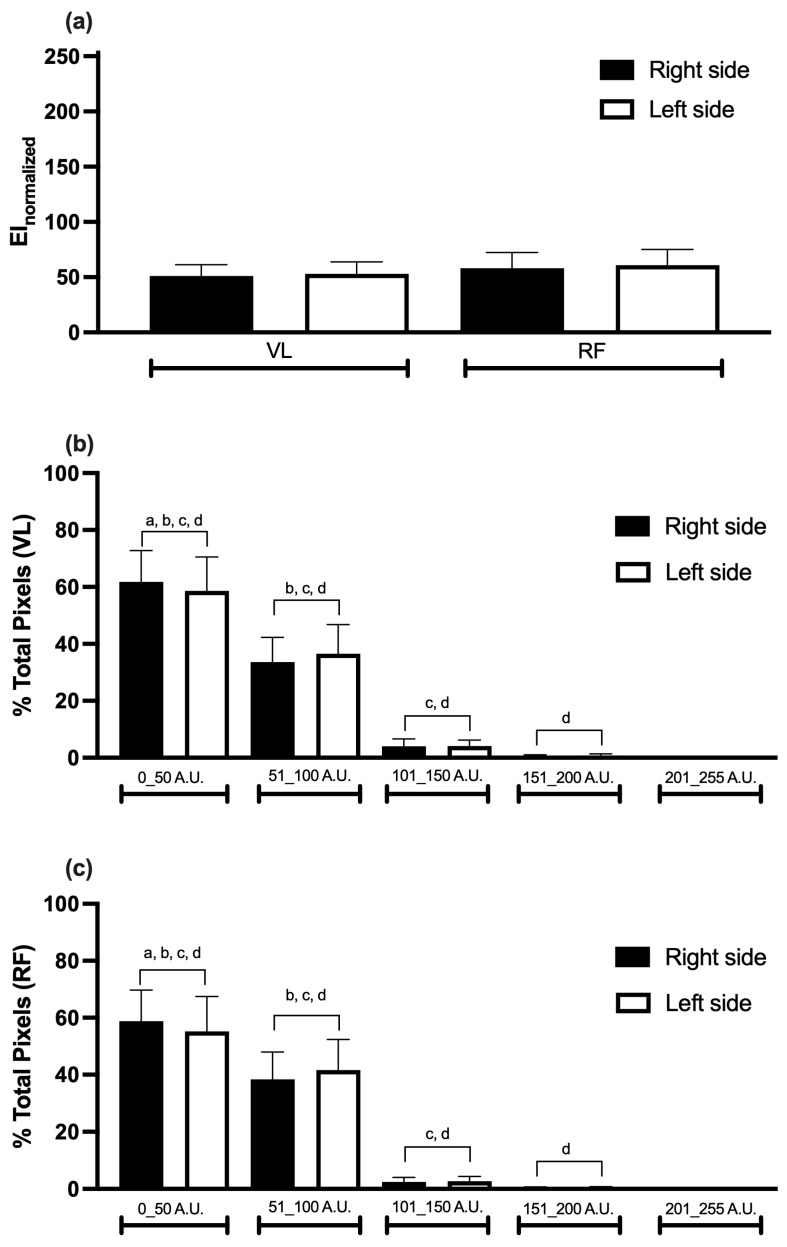
Normalized EI values for the VL and RF on the right and left sides (**a**); Distribution of pixel percentages of the VL in the different bands on the right and left sides (**b**), and Distribution of pixel percentages of the RF in the different bands on the right and left sides (**c**), where: (a) Significant difference with respect to band 51–100 A.U.; (b) Significant difference with respect to band 101–150 AU; (c) Significant difference with respect to band 151–200 A.U.; (d) Significant difference with respect to band 201–255 A.U.

Participants produced an extensor PTCon of 303.3 ± 55.3 N·m for the right knee and 289.6 ± 55.3 N·m for the left knee, with a total work of 244.8 ± 45.0 J and 228.0 ± 45.0 J, respectively. No significant differences between limbs were observed for either PTCon (*p* = 0.415, ES = 0.51) or total work (*p* = 0.228, ES = 0.62). Regarding the EI_normalized_ values (Figure 2a), the paired *t*-test revealed no significant differences between the right and left limbs for either the VL (*p* = 0.319, ES = 0.04) or the RF (*p* = 0.094, ES = 0.12). For the pixel distribution across the 5 individual spectrum bands, the analysis showed no significant interaction effect between limb sides and EI bands for either muscle group (*p* > 0.05), confirming that both right and left extremities presented a symmetrical distribution profile. However, a highly significant main effect was observed for the isolated factor “EI band” in both the VL (F(4,168) = 552.78; ηp^2^ = 0.929; *p* < 0.001) and RF (F(4,168) = 562.80; ηp^2^ = 0.931; *p* < 0.001). Post hoc Bonferroni comparisons indicated distinct pixel concentration shifts across the ranges, characterized by a progressive and significant reduction in pixel percentages as the bands transitioned toward lighter spectrum frequencies (i.e., higher attenuation values) (*p* < 0.001). Specifically, the darker 0–50 U.A. band concentrated most of the signal for both the VL (Right: ~61.8%; Left: ~58.6%) and the RF (Right: ~58.8%; Left: ~55.2%), exhibiting significantly higher percentages than all subsequent tiers (*p* < 0.001). The 51–100 A.U. range constituted the second largest zone (VL Right: ~33.6%, Left: ~36.6%; RF Right: ~38.4%, Left: ~41.6%). Conversely, the highest-intensity white pixel bands registered negligible amounts, with the 151–200 A.U. interval accounting for less than 1% of the total density, and the 201–255 U.A. tier remaining virtually absent across all evaluated soccer players (<0.05%).

Table 1 presents the coefficients of the generalized linear regression analysis, adjusted for all IE variables. For right-knee outcomes, we observed significant associations exclusively in the RF muscle. RF_EImean showed a significant negative association with both PTCon_Right (β = −4.10; 95% CI = −7.35; −0.86) and WCon_Right (β = −3.89; 95% CI = −6.34; −1.45), indicating that for each 1-unit increase in the mean EI of the RF, the PTCon of the right knee tends to decrease by 4.10 N and 3.89 W in WCon. No IE variable of the VL muscle or the other RF bands showed a significant association with the torque or concentric work outcomes in the right knee. For the left knee outcomes, we did not observe any statistically significant association with any of the IE variables investigated, including VL and RF. As shown in Table 1 the 95% confidence intervals for all regression coefficients relating to the left limb crossed the zero value.

## 4. Discussion

This study aimed to evaluate the relationship between the percentage of pixels in 5 bands of the EI histogram for the VL and RF muscles and knee extensor torque production and work. As main findings, we observed that the EImean of the RF muscle was negatively correlated with peak torque and total work. Furthermore, no significant differences were observed in EImean or across the different bands between the right and left sides for both the VL and RF. Finally, the 0–50 AU band showed the highest percentage of pixels, compared to the other bands, for both the VL and RF. Thus, the hypothesis raised in this study was refuted, since no associations were observed between the percentage of pixels in the darker bands and the greater torque production and work of the knee extensors.

The capacity for force production in the lower limbs is particularly important for the successful performance of activities of daily living and athletic performance [12]. In soccer, for example, the knee extensors play a crucial role in the execution of several skills, such as running, jumping, kicking, passing, and maintaining balance [13,14]. In this context, the assessment of strength in this muscle group among soccer players can provide information on maximal performance and lower limb biomechanics, which can be translated into common actions performed during soccer practice [15].

It is well established that multiple factors can influence muscle strength, and that aspects of muscle architecture and quality are among its determinants [16,17]. Pardo et al. [18] evaluated the relationship between the morphological characteristics of the knee extensors and torque parameters in soccer players, and they observed a positive correlation between peak torque and quadriceps muscle volume. In the same perspective, Wenzel et al. [19] evaluated athletes across different team sports and observed that muscle morphology and EI explained the variation in concentric torque of the knee extensors at 60°/s and 300°/s. These findings confirm the results of the present study, and a significant negative association was observed between the torque parameters of the knee extensors and EImean, particularly for the RF right. This association may be associated with low fat infiltration and better overall muscle quality [20]. However, the fact that it was only observed on the right side should not be discarded. This raises the hypothesis that the dominant limb of the kick may exert some influence, since most athletes reported being right-handed. However, the analyses performed did not aim to investigate the dominance of the kicking limb. We recommend that in future studies, kick dominance be considered. In addition, the absence of association between the EI variables of VL and RF with the left-sided concentric torque parameters indicates that in addition to morphological parameters, neural factors such as the pattern of recruitment of motor units may also be involved.

However, it is essential to highlight that the observed correlation occurred only in the right lower limb, which presented the highest values of torque and total work, regardless of the similarity with the left limb in relation to the EI parameters. These results can be explained by neural factors that also strongly influence force production capacity. Trezise and Blazevich [12] evaluated the anatomical and neuromuscular determinants of isokinetic force production and observed that not only morphological parameters, but mainly neural factors influenced torque production in the knee extensors. Thus, although EImeam can be an indicator of muscle quality that assists in torque production, neural factors should be considered in force production and, consequently, in the correlation evaluated in the present study.

Among the results, one observation warrants mention, although its determinants are beyond the scope of this study. Although the EImean values were similar between muscles, there was a correlation of torque only with the RF muscle. There are records in the literature about the individual influence of quadriceps muscles on knee joint torque [21]; however, this distinction was not evaluated in this study. Furthermore, the literature shows that there is heterogeneity in muscle structure, in which differences in muscle architecture [22] and EImean [23] can be observed along the entire length of the same muscle. From this perspective, the location of EImean acquisition of the VL in the present study may have been an intervening factor that contributed to the absence of a correlation with torque; however, future studies should confirm this assumption.

Regarding the EI bands, no differences were observed between the limbs, with the pixel pattern showing the highest values in the lower bands. Although soccer players have preferences regarding the use of the lower limb for task execution [24], the training program is not focused solely on a single lower limb. Thus, the absence of a significant difference in EImean may be related to the training product to which both limbs were subjected. Additionally, the pixel distribution pattern indicates that both limbs exhibit higher values in the first band (0–50 A.U.), denoting higher muscle quality, which is expected for a sample of professional players who undergo constant training and adequate dietary control.

Although the present study presents new information to the literature, some limitations should be noted. The EI assessment was performed in a single muscle region, thereby limiting the variability of muscle characteristics to a single cross-section. Information on neural aspects was not collected, which could broaden the discussions on the determinants of torque production. In addition, the brightness configuration in the ultrasound software caused the images to present a greater distribution of pixels in the darker bands, which could compromise the statistical sensitivity of bands. However, it is important to highlight that the present study evaluated a considerable sample of experienced soccer players. Additionally, studies on EI bands are still growing, and the data from this research adds information about these bands, the athlete population, and the correlations between EImean and torque and joint work.

## 5. Conclusions

We conclude that in male soccer players, only the mean echo intensity of the rectus femoris was negatively associated with knee extensor peak torque and concentric work, whereas no associations were observed for echo intensity bands or for the vastus lateralis. Echo intensity parameters did not differ between limbs, indicating bilateral symmetry. These findings suggest that mean echo intensity is more informative than band-based analysis for assessing muscle quality related to knee extensor strength in this population.

## Figures and Tables

**Table 1:** Simple linear regression analysis between right and left PTCon (peak torque concentric) and WCon (total work concentric), the EI (echo-intensity) mean values, and the % of pixels in each of the 5 bands. AIC = Akaike Information Criteria; BIC = Bayesian Information Criteria.

	PTCon_RIGHT		WCon_RIGHT		PTCon_LEFT		WCon_LEFT	
	Coef. (95% CI)	SE	Coef. (95% CI)	SE	Coef. (95% CI)	SE	Coef. (95% CI)	SE
VL_EI mean	−1.12 (−7.76; 5.65)	3.43	1.02 (−3.91; 6.08)	2.53	5.36 (−7.50; 1.75)	6.31	6.18 (−5.74; 1.74)	5.66
VL_0–50	−35.76 (−500.19; 444.95)	245.37	−59.89 (−397.34; 289.56)	179.31	280.09 (−396.20; 9.20)	327.76	129 (−519.65; 7.32)	305.38
VL_51–100	−31.83 (−492.37; 446.70)	243.30	−59.00 (−393.61; 287.56)	177.80	275.36 (−399.32; 9.14)	327.02	124.73 (−522.68; 7.26)	304.43
VL_101–150	−40.02 (−509.93; 448.57)	247.97	−69.50 (−411.17; 284.41)	181.29	274.67 (−403.81; 9.16)	329.15	122.01 (−530.52; 7.27)	306.88
VL_151–200	−98.61 (−582.44; 404.88)	256.26	−107.18 (−458.00; 256.35)	187.10	275.41 (−462.90; 9.90)	360.91	115.60 (−588.09; 7.84)	334.43
VL_201–255	na	na	na	na	na	na	na	na
RF_EI mean	−4.10 (−7.35; −0.86)	1.64	−3.89 (−6.34; −1.45)	1.26	−2.59 (−7.49; 2.17)	2.30	−3.67 (−8.41; 8.89)	2.16
RF_0–50	−1820.51 (−4763.83; 118.1)	1495.63	−1006.24 (−3204.27; 1235.06)	1118.46	−997.71 (−2252.43; 3.14)	617.84	−610.12 (−1777.59; 6.27)	564.46
RF_51–100	−1813.41 (−4759.19; 1191.05)	1496.87	−1000.38 (−3200.15; 1242.67)	1119.36	−994.10 (−2246.05; 3.15)	616.56	−606.83 (−1771.70; 6.29)	563.28
RF_101–150	−1815.86 (−4736.90; 1163.94)	1484.29	−993.54 (−3174.50; 1231.10)	1109.82	−968.28 (−2229.43; 3.47)	61.875	−580.99 (−1754.73; 6.60)	565.12
RF_151–200	−1810.62 (−4762.85; 1197.90)	1498.70	−1007.67 (−3209.87; 1236.40)	1119.80	−1202.13 (−2598.36; 2.72)	689.69	−759.96 (−2058.73; 6.32)	631.59
RF_201–255	−1674.02 (−4670.05; 1382.57)	1522.62	−858.38 (−3099.57; 1426.52)	1139.90	na	na	na	na
AIC	222.533		209.452		243.704		239.247	
BIC	236.712		223.635		256.796		252.340	

## Data Availability

The original contributions presented in this study are included in the article. Further inquiries can be directed to the corresponding author.

## Abbreviations

The following abbreviations are used in this manuscript:

EI	Echo intensity
VL	Vastus lateralis
RF	Rectus femoris
SAT	Subcutaneous adipose tissue
US	Ultrasound system
PTcon	Concentric peak torque
Wcon	Concentric total work
AIC	Akaike Information Criteria
BIC	Bayesian Information Criteria
Coef. (95% CI)	Coefficient (95% confidence interval)
SE	Standard Error
na	Non aplicable
